# Spindle cell carcinoma of the head and neck region: treatment and outcomes of 15 patients

**DOI:** 10.3332/ecancer.2015.594

**Published:** 2015-11-18

**Authors:** Muhammad Shahid Iqbal, Vinidh Paleri, Jolene Brown, Alastair Greystoke, Werner Dobrowsky, Charles Kelly, Josef Kovarik

**Affiliations:** 1 Northern Centre for Cancer Care, Freeman Hospital, Newcastle upon Tyne NE7 7DN, UK; 2 Department of Head and neck surgery, Freeman Hospital, Newcastle upon Tyne NE7 7DN, UK

**Keywords:** spindle cell carcinoma, head and neck cancer, surgery, radiotherapy

## Abstract

**Introduction:**

Spindle cell carcinoma of the head and neck is a rare entity and the evidence of optimal management is lacking. The objective of our study was to report the treatment and outcomes of 15 patients treated in a single institution over a seven year period.

**Materials and Methods:**

A total of 15 patients (12 males and 3 females) with spindle cell carcinoma of the head and neck were treated between July 2007 to June 2014. In six patients the disease developed after previous radiotherapy. Of the 15 patients, five patients had their primary in the tongue, four in the paranasal sinuses, two in the hypopharynx, two in the vocal cords, and one each in the soft palate and the floor of mouth. Eleven patients were treated with radical intent (seven patients required surgery only and four were treated with combined modality). The remaining four patients were treated with palliative intent.

**Results:**

Among 11 patients treated with radical intent eight are alive or died of non-oncological causes. The disease recurred locally in three patients and they died of the disease (two patients with locally advanced disease in the tongue and one patient with T1N0 tumour in the hypopharynx). Median overall survival (OS) was 18 months.

**Conclusion:**

Surgery or surgery combined with radiotherapy has a real impact on the natural cause of spindle cell carcinoma of the head and neck region. Even locally advanced tumours can be controlled with aggressive treatment. The worst outcome is seen with the tongue as the primary site because of a high local recurrence rate.

## Introduction

Spindle cell carcinomas (SpCC) of the head and neck are a rare variant of the squamous cell carcinoma (SCC), representing less than 3% of all head and neck malignancies of epithelial origin [1]. It is more predominant in males with a male-to-female ratio of nearly 7:1 [2]. Smoking, alcohol consumption, and previous irradiation are predisposing factors [3, 4].

Most of the available literature focuses on morphological and immunohistochemical features of this entity [5, 6, 7]. SpCC consist of elongated (spindle) cells of epithelial origin, representing an unusual form of poorly differentiated SCC though morphologically these resemble a sarcoma [8].

The most common site of origin in the head and neck region is the glottis and hypopharynx. The extra-laryngeal origin is much less frequent, although SpCC has been described in all head and neck sites. We found in six patients it even arose from the buccal mucosa [9]. In comparison with SCC, laryngeal SpCC appear to be more likely to present at an earlier stage. The metastatic potential of both entities seems to be similar [10].

Surgery is considered to be the mainstay in the management of SpCC. The effectiveness of radiotherapy was suggested by Ballo *et al* in 1998, however, more recent studies did not confirm the impact of radiotherapy on survival [11, 2]. The role of cytotoxic chemotherapy is unclear [12].

The optimal treatment of SpCC of the head and neck region is not based on high level evidence as this is a rare entity and only retrospective reports have been published.

This retrospective single institution cohort reports the management and outcome of treatment in 15 patients. In this discussion we focus on clinical aspects, especially with regards to treatment options and their outcome.

The authors are aware that analysis of 15 patients cannot answer the question of whether the management of SpCC should be different from the management of SCC of the head and neck region. However, the literature evaluating the treatment strategies of SpCC are so scarce that any contribution is of value.

This small series show an unusual site distribution of the disease. The location of SpCC of the head and neck region outside the larynx is considered to be extremely rare [3, 13]. In our local experience, five patients had their primary in the tongue/base of tongue, and four in the paranasal sinuses, and their management and outcomes add to the sparse literature in this uncommon pathological entity.

## Materials and methods

### Patient population

The patient population of histologically-proven SpCC of head and neck region treated in our institution, between July 2007 to June 2014 (seven years period) were identified from the local hospital database and the case notes were reviewed retrospectively. Medical records were analysed for age, gender, site and stage of disease, any previous radiotherapy to the head and neck region, primary and adjuvant treatment, clinical outcome and survival.

### Pre-treatment evaluation

All patients underwent pre-treatment clinical evaluation which included physical examination, upper aerodigestive tract endoscopy, biopsy under local anaesthesia ± ultrasound and needle aspiration cytology, routine blood tests and staging contrast-enhanced computed tomography scans of head, neck and chest ± magnetic resonance imaging (MRI) of neck by the otolaryngology-surgical team. All cases were discussed in Head and Neck Multidisciplinary Team (MDT) meetings comprising otolaryngologists, maxillofacial surgeons, clinical oncologists, histopathologists, radiologists, clinical nurse specialists, dietitians, and speech and language therapists. The management plans were determined on an individual basis.

### Treatment

Surgery was performed in 11 patients. Radical neck dissection was required in two patients. A complete resection (R0) was defined as complete excision of grossly visible tumour and also after the resection, margins were verified to be histologically free of tumour in a final pathological examination. An incomplete resection was defined as microscopically involved resection margins (R1) or gross residual disease (R2). Following surgery, the cases were once again discussed in MDT meetings to select the patients for adjuvant treatment. The decisions were based on final pathological stage, completion of excision, and patient’s fitness for further treatment. External beam radiotherapy was given using linear accelerator based intensity modulated radiotherapy (IMRT).

### Post-treatment follow-up

The patients treated with radical intent were followed up in the outpatient clinic on every three month basis for first two years and then every six month basis thereafter. During each visit, the patients were examined by the otolaryngology–surgical team. The evaluation included physical examination, upper aerodigestive endoscopies, and a computed tomography (CT) scan for a suspicion of recurrence.

### Statistics

The duration of disease free survival (DFS) was defined as the interval from the date of diagnosis to the date proven detection of recurrent or metastatic disease. Overall survival (OS) was defined as the duration from the date of diagnosis to the date of death or the last follow-up date. All statistical analysis were completed using statistical analysis software package SPSS (SPSS, Chicago, IL, USA).

### Ethical considerations

This retrospective study was registered with the local hospital clinical effectiveness register as a service review project. The project record number was 5748.

## Results

A total of 15 patients (12 males and 3 females) were identified during the study period. The median age was 66 years, mean age was 68 years (range: 46–84). The most common subsite was tongue/base of tongue (*n* = 5) followed by paranasal sinuses (maxilla *n* = 3; ethmoid *n* = 1). Other subsites included; two in the hypopharynx, two in the vocal cords, 1 in floor of mouth, and 1 in the soft palate. In six patients the disease developed because of previous radiotherapy, with the lag period varying widely from 1 year to 12 years (median seven years). Of these six patients, the disease developed in two patients each in the oral tongue, maxilla, and hypopharynx. Ten patients were smokers or past smokers. The details of group characteristics are summarised in Table 1.

### Single modality treatment

Seven patients were treated with surgery only. The majority of the patients in this group presented with early stage disease (Table 1) and excision was considered complete in all patients though one patient (case 2) required further excision in order to achieve the clear margins. Three patients had adverse features of lymphovascular and perineural invasion but two out of these patients did very well and both are alive with no evidence of disease recurrence after 49 and 44 months of diagnosis. Only one patient (case 4) in this group died of recurrent disease. He was an 84-year-old man who underwent surgery for pT2 base of tongue tumour. He died of recurrent disease 33 months after his diagnosis.

### Combined modality treatment

Three patients were treated with surgery followed by adjuvant radiotherapy and one with surgery and adjuvant chemoradiotherapy. Two patients (cases 8 and 9) were with early disease (pT1 of vocal cord and hypopharynx respectively) and required adjuvant radiotherapy 63 Gy in 30 daily fractions in view of microscopically involved margins (R1 resection). The patient with laryngeal disease did very well and is alive after more than five years of the initial diagnosis. However, the disease recurred in the patient with hypopharyngeal disease after 22 months of initial diagnosis and the patient died of progressive disease three months later.

The remaining two patients in this group presented with locally advanced stage IV disease. One patient (case 10) was a 78-year-old lady with T4 disease of the ethmoid required surgical resection followed by definitive chemoradiotherapy (65 Gy in 30 daily fractions with concurrent cisplatin chemotherapy 40 mg/m^2^ on a weekly basis). The patient did very well and is alive with no sign of recurrence three years after the diagnosis. The last patient in this group (case 11) presented with T4N2bM0 carcinoma of the base of tongue was managed with surgical resection followed by radical radiotherapy 65 Gy in 30 fractions. The disease recurred 18 months after the treatment and the patient died soon after with progressive disease.

### Palliative treatment

Four patients were treated with palliative intent. The first was a patient (case 12) treated originally with surgery and adjuvant radiotherapy for SpCC of the oropharynx seven years prior to the diagnosis of SpCC. Local recurrence and distant metastases developed two years before the diagnosis of SpCC and was treated with palliative chemotherapy with 5FU/carboplatin and cetuximab with partial response. Further progression in maxillary alveolus was resected and histology revealed features of SpCC. The patient died of progressive disease 12 months later. Another patient (case 15) with T4N0M0 SpCC of the maxilla was deemed medically unfit for surgery and treated with palliative radiotherapy but died after five months of progressive disease.

The remaining two patients were managed with best supportive care. One patient (case 13) presented with a T2N2bM0 SCC of the base of tongue and died three months after the biopsy. One patient (case 14), a female patient originally presented with SCC pT4bN0M0 of the maxilla and was treated with surgery and adjuvant chemoradiotherapy. Progressive disease developed eight months after the treatment and the diagnosis of SpCC was histologically verified. The patient was allocated for best supportive care and died one month after the biopsy.

### Prognostic factors affecting the outcome

The median follow-up time at the time of analysis was 18 months (range: 1–62). Of the total 15 patients, 10 patients had died. A statistical analysis was carried out to find out the prognostic factors affecting the outcome. Using multivariate analysis, nodal involvement (N0; N2/3, *p* = 0.029) and treatment modality (surgery; surgery and adjuvant treatment; palliative management, *p* = 0.038) were found to be the prognostic factors affecting the outcome. However, age (<70; >70, *p* = 0.38), T stage (1; 2; 3; 4, *p* = 0.29), disease site (*p* = 0.79), extent of excision (complete; incomplete, *p* = 0.72) and the presence of lymphovascular invasion (*p* = 0.43) had no statistically significant impact on survival outcome.

## Discussion

Traditionally, surgery is considered as the mainstay treatment of the SpCC. This fact is explainable by the location and natural history of the disease. The majority (70%) of the SpCC are located in the glottic region and present with symptoms of hoarseness, dyspnoea and cough, appearing as polypoidal and pedunculated masses, usually less than 2 cm in size [14]. In this situation, a wide local excision is a radical treatment as the underlying stroma is not involved by the tumour. The outcome of surgery alone for early SpCC is excellent. More advanced disease requires combined treatment with adjuvant radiotherapy. SpCC were traditionally believed to be aggressive, radioresistant tumours, and radiotherapy was considered to have a limited role [15].

Ballo *et al* reviewed 28 cases of early sarcomatoid carcinoma of the larynx (21 were classified as T1N0M0 and 7 as T2N0M0) treated with radical radiotherapy (mean dose 65 Gy). With the median follow-up of ten years (range from 1.5 to 24 years) only four patients recurred and were salvaged with radical laryngectomy. The study concluded that there was no difference in outcome of radiotherapy between SCC and SpCC and that the histologic diagnosis of sarcomatoid carcinoma by itself should not influence the decision to treat a patient with early stage glottic disease with irradiation [11].

Ballo’s observation has not been confirmed by a recent study by Dubal *et al* who analysed 312 cases of laryngeal SpCC based on extraction from the Surveillance, Epidemiology, and End Results (SEER) registry. All patients with SpCC diagnosed between 1973 and 2011 were included in the study. The best outcome was seen in cases when surgery was used in combination with radiotherapy

(five year disease specific survival, DSS, of 84.2%) although this DSS rate was almost similar to cases when surgery was used alone without radiotherapy (84.0%). The study concluded that surgery was independently associated with better outcome (five year DSS of 84.1% in cases when any surgery was utilised compared to 57.1% in cases when surgery was not used, *P* = 0.0003). Also, the role of radiotherapy was analysed. Radiotherapy did not alter the prognosis (five year DSS of 75.6% in cases when radiotherapy was utilised compared to 75.8 in cases when radiotherapy was not used, *P* = 0.7959). In cases, when radiotherapy was the sole modality of treatment, the outcome was least favourable with a five-year DSS of 60.5%. However, the authors admitted that these cases probably represented more advanced disease where surgery was not feasible. In this study, no details of radiotherapy (total dose, radiotherapy technique) were provided. It is likely that radiotherapy was indicated in more advanced cases even in adjuvant settings [2].

Thompson *et al* reviewed 187 cases of laryngeal SpCC. Seventeen patients (9%) had a history of a prior radiation of the larynx. All patients were managed by surgery (excisional biopsy only in 24 patients, excisional biopsy followed by radical surgery in 66 patients and surgery followed by adjuvant radiotherapy in 97 patients). This study, as most of the literature focused on diagnostic controversies (histopathological features) and clinical aspects were not reported in detail. The dose of radiation ranged from 2–72Gy (incomplete treatment course to full treatment). The authors relied on the records of the Data Oncology Services (cancer registry) of referring departments. They also reported a lower percentage of patients who died of their disease in the surgery only group (18.5%) compared with the surgery and radiation group (42.3%), although the survival was longer in the radiation group (3.6 years) compared to those in the surgery only group (1.9 years). There was no significant difference in length of survival between the patients managed by radiation alone (no salvage, mean survival 3.9 years) versus patients who had radiation and a salvage procedure (mean survival 3.9 years). The authors conclude that patients who were managed with surgery alone had a better outcome than patients managed with surgery and postoperative radiation. However, the authors admitted that they were unable to make a specific comment about the possibility that radiation therapy was used only in the more clinically advanced cases. It is not possible to draw any conclusion concerning the role of radiotherapy, and also it is not possible to recommend the dosage of radiotherapy or the volume to be treated [6].

Two retrospective series showed poor survival outcomes. In the series by Benninger *et al*, 13 out of a total 15 patients died of progressive disease despite aggressive management with an average of 14.5 months after presentation. Oral cavity and sinonasal carcinomas, in particular had poorer prognosis than SCCs at similar sites [16]. Su *et al* reported outcome on 18 patients of SpCC of oral cavity and oropharynx. The median OS was 8.9 months with three years survival rate was 100% in early stage group (stage I & II) and for stage III and IV, one year survival was 9% and three year was 0% respectively [8].

Vazquez *et al* compared the outcome and treatment of various variants of sinonasal carcinoma and concluded that SpCC was more aggressive than that of conventional SCC with worse prognosis despite aggressive treatment. Using SEER database, the authors compared 4382 cases of conventional sinonasal SCCs with 328 cases of its major variants. Five year DSS for verrucous cell carcinoma was 84.7%, 61.87% for papillary cell carcinoma, 56.2% for basaloid cell carcinoma, 45% for conventional squamous cell, 32% SpCC, and 15% for adenosquamous carcinoma. In the same time, more patients with SpCC were treated with combination of surgery and radiotherapy (65.6%) than patients with conventional SCC (40.4%) [17].

Reys *et al* reported two cases of SpCC of the tongue. Both patients presented with locally advanced disease (T4N2M0 and T4aN1M0) and were managed with a combined modality treatment i.e. radical surgery followed by adjuvant chemoradiotherapy. Pulmonary metastasis developed in first patient in five months after surgery while the second patient was disease-free two months after surgery [18].

Our analysis confirmed unfavourable prognosis of extralaryngeal SpCC of the head and neck region. Two patients with laryngeal involvement were diagnosed with early stage disease and cured. Otherwise we can agree with the observation of Bice *et al*, who found survival of SpCC of the oral cavity and oropharynx worse than survival of conventional SCC. At the same time, there was no difference in the outcome for larynx or hypopharynx site. In his large study analysing 118 patients he identified in multivariate analysis only age, tumour size, and M (metastatic) stage as variables significantly affecting the survival with SpCC [19].

**Figure 1.**
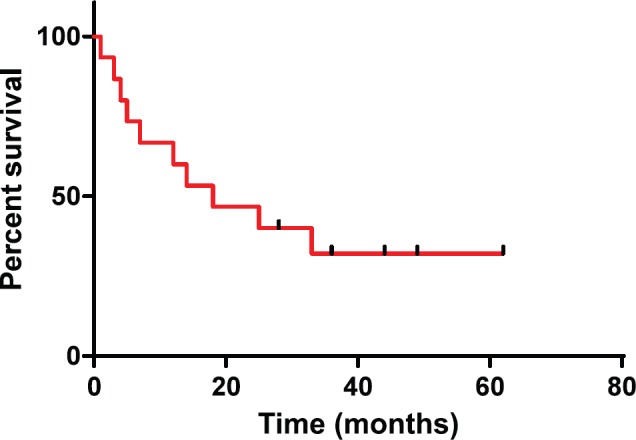
Overall survival probabilities were estimated using Kaplan-Meier curves.

Our series illustrated better outcome as compared to that of Benninger *et al*, Su *et al* and Reys *et al*. The median OS in our study was 18 months (Figure 1). This may be explained because of the improved surgical techniques, obtaining clear margins, and employment of adjuvant (chemo)radiotherapy in cases where surgical margins were unclear or in high risk disease. A comparative finding of the SpCC of head and neck region studies are summarised in Table 2.

Certain hope in the treatment of SpCC is the employment of epidermal growth factor receptor (EGFR)-specific therapies. As EGFR is expressed by >90% of conventional SCC and anti-EGFR therapies are commonly used to treat head and neck SCCs. Limited data in this regard are available for SpCC. Watson *et al* investigated 30 cases of SpCC and found that EGFR was expressed in 21/30 (70%) cases, including in the squamous component in 18/19 (95%) and the spindle cell component in only 12/30 (40%). Where the spindle cell component was positive, the intensity and distribution was lower than that for squamous component. Survival analysis did not reveal statistically significant differences in overall or disease free survival between the EGFR expressing or non-expressing groups. The authors concluded that EGFR-specific therapies may not be ideal for SpCC patients [20].

## Conclusion

The authors concede that based on a retrospective series of 15 cases, no definitive conclusions can be inferred but in view of the rarity of the disease, it is impossible to generate higher level evidence. Based on our experience, we recommend that SpCC of the head and neck region should be treated aggressively with surgery and adjuvant (chemo)radiotherapy should be considered in all cases with high risk features i.e. stage III and IV, positive/unclear margins, extracapsular spread and vascular/perineural invasion. Tumours located in the tongue are associated with unfavourable prognosis despite aggressive treatment.

## Conflict of interest

None declared.

## Figures and Tables

**Table 1.** A summary of the patient and tumour characteristics.

Case Number	Age	Gender	Date of diagnosis	Site	Stage	Primary treatment	Resection outcome	Adjuvant treatment	DFS (months)	Alive/ Dead	OS (months)	Comments
**Surgery only**												
1	71	M	02/02/2008	FOM	T1N0M0	surgery	R0	Nil	4	D	4	Died of cardiac failure
2^*^	65	M	29/11/2010	Hypopharynx	T3N0M0	surgery	R0+LVI PNI	Nil	49	A	Alive	No recurrence
3^*^	60	M	30/04/2011	Tongue	T2N0M0	surgery ND	R0	Nil	44	A	Alive	No recurrence
4^*^	84	M	29/07/2011	BOT	T2N0M0	surgery	R0	Nil	33	D	33	Died of recurrent disease
5	63	M	02/04/2012	Tongue	T1N0M0	surgery	R0	Nil	14	D	14	Died of lung cancer
6	79	M	24/08/2012	Vocal cord	T1N0M0	surgery	R0	Nil	28	A	Alive	No recurrence
7	54	M	08/04/2014	Soft palate	T4aN3M0	surgery ND	R0 LVI PNI	Not fit	7	D	7	Died of pneumonia
**Surgery + adjuvant (chemo)radiotherapy**												
8	82	M	28/10/2009	Vocal cord	T1N0M0	surgery	R1	63 Gy RT	62	A	Alive	No recurrence
9^*^	63	M	18/11/2011	Hypopharynx	T1N0M0	surgery	R1	63 Gy RT	22	D	25	Died of PD
10	78	F	12/12/2011	Ethmoid	T4N0M0	surgery	R2	65 Gy CRT	36	A	Alive	No recurrence
11	64	M	02/02/2012	BOT	T4N2bM0	surgery	R2	65 Gy RT	18	D	18	Died of PD
**Palliative management**												
12^*^	66	M	17/11/2011	Maxilla	Recurrence	cetuximab	N/A	N/A	0	D	12	Died of PD
13	68	M	20/11/2012	BOT	T2N2bM0	Nil‡	N/A	N/A	0	D	3	Died of PD
14^*^	75	F	08/03/2013	Maxilla	Recurrence	BSC	N/A	N/A	0	D	1	Died of PD
15	46	F	20/07/2013	Maxilla	T4N0M0	pall RT	N/A	N/A	0	D	5	Died of PD

Abbreviations used: FOM = floor of mouth; BOT = base of tongue; ND = neck dissection; LVI = lymphovascular invasion; PNI = perineural invasion; DFS = disease free survival; OS = overall survival; D = dead; A = alive; CRT = chemoradiotherapy; PD = progressive disease; pall = palliative; RT = radiotherapy; BSC = best supportive care; pall RT = palliative radiotherapy; N/A = not applicable. The patients with

* received radiotherapy to head neck region in the past.

+The surgical margins were positive on first surgery and required further excision.

‡ The patient deteriorated prior to palliative radiotherapy.

**Table 2.** A summary table of the SpCC of head and neck region studies.

Study	Number of patients	Sites	Treatment	Outcome/comments
Su *et al *2006 [8]	18	OC and OPX only Tongue 28% Buccal mucosa 22%	Surgery (*n* = 15) Chemo only (*n* = 1) RT only (*n* = 1) No treatment (*n* = 1)	Median OS 8.9 months 3-yr survival rate was 100% in early stage group (stage I & II) In stage III & IV, 1-yr survival was 9% and 3-yr was 0%
Thompson *et al *2002 [6]	187	Larynx only Glottic 71% (T1 59%)	Surgery (*n* = 90) Surgery + RT (*n* = 97)	17 patients had previous radiation exposure Overall raw 5-year survival was 58.8% T stage (*p* < 0.007), tumour location (glottic vs other location; *p* < 0.001), vocal cord mobility (*p* = 0.013), previous irradiation (*p* = 0.013) and necrosis (*p* = 0.017) were significant factors
Ballo *et al *1998 [11]	28	Glottis (T1, T2) only T1 = 75% T2 = 25%	RT only median dose 65 Gy	Median follow up 10 years 4 patients had disease recurrences in larynx and required salvage laryngectomies 5-year actuarial LC rate for T1 and T2 patients were 94% and 54% respectively
Benninger *et al *1992 [16]	15	OC 40% Larynx 20% OPX 20% Maxillary sinus 20%	Surgery (*n* = 13) (including surgery + RT *n* = 5) Unresectable disease (*n* = 2)	13 out of 15 patients (87%) died of their disease with an average of 14.5 months after presentation
Olsen *et al *1997 [14]	34	Larynx 74% (T1 glottic *n* = 16) Hypopharynx 26%	Surgery (*n* = 30) Biopsy only (*n* = 2) Surgery + RT (*n* = 1) RT only (*n* = 1)	The median follow up of 31 patients was 3.7 years Disease recurred in 10 patients (29%) The estimated of surviving at least 3 years after initial treatment was 56.8% 3-year survival rate for the patients with laryngeal tumours was 76.2%
Gerry *et al *2014 [10]	341	Larynx 46% OC 20% OPX 18%	Surgery only (*n* = 77) RT only (*n* = 99) Surgery + RT (*n* = 130)	A retrospective comparative analysis from SEER database of SpCC to conventional SCC SpCCs were more likely to be high grade (*P* > .001) and present at an early stage (*P* < .001 to *P* < .05) Distant metastasis rates were similar between the tumor types DSS was similar between SpCCs and SCCs, although site-specific survival rates were higher for SpCCs of larynx (*P* = .017) and lower for those of OC (*P* = .008)
Vazquez *et al *2014 [17]	110	Sinonasal	Surgery + RT = 65.6% of total patients with SpCC	A retrospective comparative analysis from SEER database of sinonasal SCC to its variants 5-year DSS for verrucous cell carcinoma was 84.7%, 61.87% for papillary cell carcinoma, 56.2% for basaloid cell carcinoma, 45% for conventional squamous cell, 32% SpCC and 15% for adenosquamous carcinoma
Dubal *et al *2015 [2]	312	Larynx	Surgery (*n* = 200) No surgery (*n* = 102) Radiotherapy (*n* = 224) No radiotherapy (*n* = 82)	A retrospective analysis from SEER database (1973–2011) 1, 5, 10-year relative survival rates were 91%, 77.7% and 64.5% respectively Primary glottis tumours had a favourable prognosis than that of nonglottic tumours (5-year DSS of 84% verse 51.9%) Radiotherapy did not alter survival rates
Our study	15	Tongue/BOT 30% Paranasal sinuses 26% Hypopharynx 13% Vocal cord 13% Mandible 1% Soft palate 1%	Surgery only (*n* = 7) Surgery + RT (*n* = 3) Surgery + CRT (*n* = 1) Palliative (*n* = 4)	Median OS was 18 months 5 of 11 patients treated with radical intent are alive Median OS for patients treated with radical intent was 28 months Nodal involvement (N0; N2/3, *p* = 0.029) and treatment modality (surgery; surgery and adjuvant treatment; palliative management, *p* = 0.038) were found to be the prognostic factors affecting the outcome

Abbreviations used: OC = oral cavity; OPX = oropharynx; RT = radiotherapy; chemo = chemotherapy; OS = overall survival; LC = local control; SCC = squamous cell carcinoma; DSS = disease specific survival; BOT = base of tongue; CRT = chemoradiotherapy.
